# RETRACTION: Polo‐Like Kinase 4 Correlates With Greater Tumor Size, Lymph Node Metastasis, and Confers Poor Survival in Non‐Small Cell Lung Cancer

**DOI:** 10.1002/jcla.70241

**Published:** 2026-04-13

## Abstract

**RETRACTION**: Q. Zhou, G. Fan, Y. Dong, “Polo‐Like Kinase 4 Correlates With Greater Tumor Size, Lymph Node Metastasis, and Confers Poor Survival in Non‐Small Cell Lung Cancer,” *Journal of Clinical and Laboratory Analysis* 34, no. 4 (2019): e23152, https://doi.org/10.1002/jcla.23152.

The above article, published online on 25 December 2019 in Wiley Online Library (wileyonlinelibrary.com), has been retracted by agreement between the journal Editor‐in‐Chief, Rong Fu; and Wiley Periodicals, LLC. The retraction has been agreed due to concerns raised by a third party, which identified compromised data and systematic issues in the published article. The editors have lost trust in the reliability of the data and the results presented. The corresponding author confirmed scientific problems and requested retraction.

**RETRACTION**: Q. Zhou, G. Fan, Y. Dong, “Polo‐Like Kinase 4 Correlates With Greater Tumor Size, Lymph Node Metastasis, and Confers Poor Survival in Non‐Small Cell Lung Cancer,” *Journal of Clinical and Laboratory Analysis* 34, no. 4 (2019): e23152, https://doi.org/10.1002/jcla.23152.

The above article, published online on 25 December 2019 in Wiley Online Library (wileyonlinelibrary.com), has been retracted by agreement between the journal Editor‐in‐Chief, Rong Fu; and Wiley Periodicals, LLC. The retraction has been agreed due to concerns raised by a third party, which identified compromised data and systematic issues in the published article. The editors have lost trust in the reliability of the data and the results presented. The corresponding author confirmed scientific problems and requested retraction.

